# Nutritional status and dietary diversity of pregnant women in rural KwaZulu-Natal, South Africa

**DOI:** 10.4102/hsag.v24i0.1114

**Published:** 2019-10-23

**Authors:** Carin Napier, Kelly Warriner, Maureen N. Sibiya, Poovendhree Reddy

**Affiliations:** 1 Department of Food and Nutrition Consumer Science, Durban University of Technology, Durban, South Africa; 2 Department of Nursing, Faculty of Health Science, Durban University of Technology, Durban, South Africa; 3 Department of Community Health Studies, Faculty of Health Sciences, Durban University of Technology, Durban, South Africa

**Keywords:** pregnancy, nutritional status, food diversity, diet, first trimester of pregnancy, nutritional status

## Abstract

**Background:**

Pregnancy is a critical period during which maternal nutrient intake and nutritional status impact both the mother and the infant. Various factors including good nutrition play a role in a healthy pregnancy outcome. A healthy diet has an important role in the birthweight and well-being of both the mother and the child.

**Aim:**

The aim of this descriptive study was to determine the nutritional status and food intake of a group of pregnant women (*N* = 100) in early pregnancy (up to 24 weeks gestation).

**Setting:**

The study took place in a Public Health Care Facility located at Umkhumbane (Mayville) and forms part of the EThekwini district operated by the Provincial and eThekwini Municipality situated in Kwa Zulu Natal, South Africa.

**Methods:**

This study utilised a quantitative, descriptive research design and included 100 pregnant women attending a public healthcare clinic in KwaZulu-Natal province, South Africa. Consenting women were measured for height and weight to determine body mass index (BMI) as an indicator of nutritional status. Food intake was evaluated through two 24-h dietary recall questionnaires and a food frequency questionnaire. Actual food intake was analysed for nutrient content and compared to the Dietary Reference Intake for women aged 19–30 years. A food variety score and food group diversity score were determined to establish the adequacy of the diet to support the first phase of pregnancy.

**Results:**

Except for carbohydrates and vitamin A, all the nutrients consumed by the women were lower than the recommended daily amounts. Fruit and vegetable intake was half of the recommended daily amount and a medium food variety score was observed. A large percentage (55%) of the women had a BMI that fell in the obese category.

**Conclusion:**

Although various factors can impact birth outcome, food choices made by women did not reflect the food choices to maintain a healthy pregnancy and contribute to a healthy birth outcome. Nutrition education aimed at girls of childbearing age and pregnant women is important to increase their awareness about a healthy pregnancy and healthy birth outcome.

## Introduction

Women living in developing countries are more often exposed to food insecurity and malnutrition, which are two of the leading causes of maternal and neonatal morbidity as well as other long-term effects that can affect the growth and development of the foetus (Misselhorn & Hendricks 2017). Inadequate diet and/or insufficient access to food is said to have a critical role in the overall health status of the mother and foetus as both under- and over-nutrition can have a serious impact on the long-term health status and life expectancy of the mother and foetus (Kavle & Landry 2018; Zeisel 2011). Furthermore, prenatal malnutrition can have lasting effects on children as both micro- and macronutrients play a vital role in the health outcome of the foetus and are necessary to ensure maternal health throughout the pregnancy (Zeisel 2011). Moreover, during pregnancy, it is essential that a nutrient-rich diet is consumed to meet specific requirements of macro- and micronutrients, including energy, protein, calcium, folic acid, iron, zinc and iodine, to name a few (Zeisel 2011; Zerfu & Ayele 2013).

In developing countries like South Africa, a quality diet has traditionally been equated with sufficient intake of energy and essential nutrients. Diets more concentrated in sugars, saturated fats, salt and processed cereals still seem to be regularly consumed by pregnant women despite efforts to educate them and encourage about a healthier, more balanced diet (Misselhorn & Hendricks 2017). Undernourished women at the time of conception and throughout the pregnancy are at increased risk of experiencing adverse pregnancy outcomes, such as spontaneous abortion, preterm birth, foetal growth restriction and hypertensive disorders (Müller & Krawinkel 2005; Ramakrishnan et al. 2012). In addition, women experiencing under-nutrition during earlier stages of pregnancy are at an increased risk of metabolic disorders and complications during labour and birth. It has been reported that adequate maternal weight gain during the last two trimesters of pregnancy is essential to support the growth of the foetus (Nguyen 2019). However, weight gain may not be directly correlated with adequate nutrition as reflected by dietary intake and few studies have explored the relationship between weight gain and maternal nutrition (Kavle & Landry 2018). This study aimed to determine the nutritional status and dietary diversity of a group of pregnant women in early pregnancy in KwaZulu-Natal (KZN) province of South Africa.

## Research methods

The research design utilised a prospective descriptive approach examining a subset of data from a larger birth cohort study. The larger study was designed to evaluate and improve antenatal care (ANC) access and clinic attendance at the primary healthcare (PHC) level in KZN.

### Participants and sampling

The study population included pregnant women who presented for their first ANC visit at the PHC before 24 weeks of pregnancy and who consented to participate in the study. A convenience sample comprising 100 pregnant women was enrolled into the study. Inclusion criteria were as follows: women over 18 years of age, with the ability to read and speak isiZulu or English. Gestational age was confirmed by their last normal menstrual period and physical examination by an experienced midwife. Women were excluded from the study if they were diagnosed with a chronic health problem (e.g. diabetes, thyroid disease, chronic hypertension, sickle cell disease and HIV and AIDS), were taking regular medications other than prenatal vitamins and folic acid, or had multiple gestations. Recruitment and data collection occurred between October and December 2015. Ethical clearance for the study was obtained from the Institutional Research Ethics Committee, Durban University of Technology (IREC 045/14) and the KZN Department of Health (HRKM 234/14). All participants gave written informed consent prior to study enrolment.

### Setting

Participants in this study entered prenatal care at a public PHC clinic, which provided services to a resource poor community in the eThekwini District, KZN. The clinic typically schedules pregnant women to be seen according to their gestational age and provides folic acid supplements on entry into care. According to Pattinson (2005), every site where pregnant women make contact with health services should provide daily basic antenatal care (BANC) services so that the first ANC consultation takes place as soon as the pregnancy has been confirmed or the very first time that a pregnant woman visits a health facility. However, the South African BANC Guidelines recommend a minimum of four ANC visits during pregnancy. A nutrition adviser was available at the clinic where the study was conducted.

### Data collection

All participants were weighed in a private room using an electronic calibrated medical scale and their height was measured using a standard stadiometer. Body mass index (BMI) was subsequently calibrated at the first prenatal visit. Body weight, in light clothing with no shoes, was determined to the nearest 0.1 kg on a newly calibrated portable electronic physician scale (Portable Physician Scale [PPS] Scales 2000 – SA). Each group was weighed and measured by the same fieldworker to ensure consistency in the measuring method. Height was measured to the nearest 0.5 cm using a portable stadiometer (PPS Scales 2000 – SA). All measurements were taken twice and the average of the two measurements was recorded if a difference was observed.

A full health and sociodemographic profile was collected for each participant. All four administered questionnaires were previously tested for validity and reliability. An expert focus group was convened to ensure validity of all data collection instruments, while reliability was tested by piloting all questionnaires prior to the study. Fieldworkers, who were fluent in both English and isiZulu, assisted the participants to complete the food intake questionnaires as very specific information was required with respect to daily food intake, frequency and portion sizes of meals. Two validated 24-h recall questionnaires were administered – one reporting on food intake on a week day and one reporting on food intake on a weekend day– to determine the actual food items that were consumed by the participants, including the portion sizes.

A validated food frequency questionnaire (FFQ) was adapted after the pilot study to accommodate all possible food items in the nine nutritious food groups as identified by the Food and Agriculture Organisation (FAO) (Food and Agriculture Organization 2011) that could be consumed by this population group. The FFQ requires participants to specify what food items have been consumed within the last 7 days, selecting from a variety of food options from various food groups. These food groups include flesh foods (meat and poultry), eggs, dairy products, cereals, roots and tubers, legumes and nuts, vitamin A rich fruit and vegetables, other fruits, juices, other vegetables and fats and oils. A low food variety score (FVS) was indicated by less than 30 foods consumed in a period of 7 days, and a medium FVS was indicated by 30–60 foods, while a high FVS indicated more than 60 foods consumed in the same period. A high food group diversity score (FGDS) represented the consumption of food from more than six food groups, and a medium FGDS between four to five food groups and consumption of less than three food groups was shown as a low FGDS.

### Data analysis

Data analysis was completed using MS Excel^®^ (version 14). The BMI was calculated using the weight (kg) of each participant divided by height squared (m²) and compared to World Health Organization (WHO)-published BMI guidelines for women (World Health Organization 1995), which is the same as for women in the first trimester of pregnancy. The 24-h recall questionnaires were captured and analysed using the Food Finder 3 software of the South African Medical Research Council (SAMRC) (Wolmarans et al. 2010). Fortified maize and bread were added manually to the Food Finder program before the analysis was conducted, and the data were presented as means and standard deviations (SDs) and compared with the Dietary Reference Intake (DRI) of the National Academy of Sciences (Nutrition Information Centre University of Stellenbosch 2017a). Frequency intake and per capita intake were also calculated and presented as the top 20 foods consumed by the group. The FFQs were analysed using the Statistical Program for Social Sciences (IBM Corp, IBM SPSS Statistics for Windows, version 23.0. Armonk, NY).

The FFQs were coded and presented in terms of frequencies, percentages, mean and SD for the various categories to indicate the FVS and FGDS (FAO 2011).

### Ethical considerations

The part of the overall Medical Research Council study that is reported in this article was approved by the Durban University of Technology (DUT) ethics committee (ethical approval number: IREC 017/15). The participants of the study were Zulu-speaking people and their literacy levels were unknown; therefore, Zulu-speaking fieldworkers were used to explain the study, for data collection and to assist with the completion of questionnaires.

Permission to conduct the study at the Cato Manor Community Health Centre in Umkhumbane was obtained from the Department of Health. Then permission was obtained from the clinic manager. All the participants meeting the inclusion criteria at the selected clinic were approached until the sample requirement of 100 participants had been reached. The study was explained to all the participants and a written consent form was obtained from each participant before the study commenced. The participants understood that data would be collected throughout the pregnancy and that they could withdraw from the study at any time with no consequences. The participants were assured of confidentiality and anonymity of information. A number code was allocated to each participant.

## Results

### Population characteristics

Pregnant women (*N* = 100) who participated in the study were black Africans, with an average gestational age on study entry of 15.44 weeks (SD = 5.31; range 8–24). The mean maternal age was 25.73 years and most had completed secondary schooling (72.70%), with 4% (*n* = 4) having no formal education. It was found that 65.00% (*n* = 65) of the women were unemployed, while 67.2% (*n* = 43) reported an income of less than R1500 per month. The majority of the households (90.00%) had access to running water in the household or yard, 70% had flush toilets and 85% had access to waste removal services in their community. Although the majority (57%) of the households had two to five people and 10% had six and more people living in it, 65.3% of the households seldom or never had money to purchase food (Table 1).

**TABLE 1:** Socioeconomic profile of the pregnant women (*n* = 100).

Variable	Socioeconomic indicators	*n*	%
**Educational profile**	No formal	4	4.00
	Primary	6	6.10
	Secondary	73	72.70
	College or FET	11	11.10
	Other	6	6.10
**Income of the household**	< R1500	43	43.00
	R1501–R3000	10	10.00
	R3001–R5000	5	5.00
	R5001+	6	6.00
	Declined to answer	36	36.00
**Work status**	Unemployed	65	65.00
	Employed	35	35.00
**Access to running water**	Tap in house	39	39.00
	Tap in the yard	51	51.00
	Borehole	1	1.00
	Fetch water elsewhere	9	9.00
**Toilet facilities**	None	3	3.00
	Pit latrine	26	26.00
	Flush toilet	70	70.00
	Bucket system	1	1.00
**Waste removal services**	Yes	85	85.00
	No	15	15.00
**Number of people living in the house**	1 person	31	31.00
	2–5 people	57	57.00
	6–8 people	10	10.00
	9 or more	2	2.00
**Frequency of no money for food**	Always	2	2.00
	Often	7	7.00
	Sometimes	24	24.00
	Seldom	23	23.00
	Never	39	39.00
	Unknown	5	5.00
**Average amount spent on food per month**	< R500	23	23.00
	> R500	40	40.00
	Did not know	37	37.00

FET, Further Education and Training; R, Rand.

### Body mass index

The mean height was 167 cm (SD ± 0.99 cm) and the mean weight was 67.10 kg (SD ± 14.85 kg). The mean BMI was 27.09 kg/m^2^ (SD ± 6.34 kg/m^2^). Two of the women were underweight (BMI < 18.5), 40 had a normal BMI (18.5–24.5) for a pregnant woman in the first trimester of pregnancy, and 28 were overweight (BMI 25–25.9), 20 were obese class 1 (BMI 30–34.9) and 7 were obese class 2 (BMI 35–39.9), presenting a total of 55 of the women classified as overweight and obese (Table 2).

**TABLE 2:** Body mass index classification for pregnant women (*N* = 100).

BMI classification†	*n*	%
Underweight < 18.5	2	2
Normal 18.5–24.9	40	40
Overweight 25–29.9	28	28
Obese I 30–34.9	20	20
Obese II 35–39.9	7	7
Morbidly obese > 40	3	3
**Total**	**100**	**-**

BMI, body mass index.

† , BMI classification based on WHO guidelines 1995.

### Nutrient intake

The mean energy intake of women was 58.04% (5772.71 kJ) of the DRI for energy (9926 kJ), with 95% of the women consuming less than 100% of the DRI even though the mean carbohydrate intake exceeded the required amount by 42% and 16% of the women did not consume 100% of the 135 g required per day. The women consumed a mean of 15.05 g fibre. The mean intake of protein for the group was 50.4 g (71.0%) of the DRI of 71 g. The women’s mean intake of vitamin A was above the recommended intake of 550 µg at 643.53 µg; however, 79% of the women did not reach 100% of the DRI. Ninety-eight per cent and 32% of the women consumed less than 100% of the DRI for iron (mean intake of 10.70 mg of 22 mg) and zinc (mean intake of 9.02 mg of 9.5 mg). Folate from dietary sources contributed a mean of 52.08% of the DRI for folate, with 74% of the women not meeting the DRI of 520 µg. While all women were given folic acid supplements at ANC visits, compliance in taking these supplements was uncertain.

The top three food items consumed, as shown in the top 20 foods consumed list presented in Table 4 and ranked by frequency, were all starch-based and above the recommended carbohydrate portion intake. Food contributing to protein intake included chicken, egg and beef curry ranked at 4th, 13th and 14th in the top 20 foods ingested, which correlates with the low-protein contribution to the diet indicated in Table 3. Fruit and vegetables appeared at 8th, 9th, 15th, 16th, 18th and 19th on the top 20 list, which explains the presence of the variety of fruit and vegetables in the FVS; however, the per capita intake for fruit and vegetables was 165.73 g (SD ± 64.51 g) less than half of the > 400 g recommended by the World Health Organization (2003).

**TABLE 3:** Nutrient intake as measured by two 24-h recall questionnaires compared to the daily recommended intake (*N* = 100).

Nutrients per day	Mean ± SD	Mean % of the DRIs	% of women consuming <100% of DRIs	*n*	DRI
Energy (kJ)	5772.72 ± 2057.88	58.00	95.00	95	9946 EER
Total protein (g)	50.44 ± 20.44	71.00	84.00	84	71 RDA
Carbohydrates (g)	191.82 ± 68.72	142.10	16.00	16	135 EAR
Total dietary fibre (g)	15.05 ± 6.22	53.70	98.00	98	28 AI
Vitamin A (µg)	643.53 ± 1450.35	117.00	79.00	79	550 EAR
Iron (mg)	10.70 ± 4.17	48.70	98.00	98	22 EAR
Zinc (mg)	9.02 ± 4.38	94.98	32.00	32	9.5 EAR
Folate (µg)	270.83 ± 129.38	52.08	74.00	74	520 EAR
Magnesium (mg)	170.53 ± 57.67	58.80	96.00	96	290 EAR
Riboflavin (mg)	0.99 ± 0.89	82.78	61.00	61	1.2 EAR
Calcium (mg)	308.86 ± 235.24	30.81	98.00	98	1000 AI

EER, estimated energy requirements (Institute of Medicine 2003); EAR, estimated average requirements; AI, adequate intake, used where EAR is not available; RDA, recommended dietary allowance; DRI, Dietary Reference Intake.

**TABLE 4:** Top 20 food items ranked by reported intake as measured by two 24-h recall questionnaires (*N* = 100).

No.	Food item	Mean intake per day (g)	Frequency (per day)	Portion size per frequency (g)	Per capita intake (g)
**1**	Bread/Rolls	10 314	84	122.79	103.14
**2**	Rice	10 012.5	73	138.10	100.13
**3**	Maize meal	11 790	66	178.64	117.90
**4**	Chicken	4987.5	38	133.00	49.88
**5**	Cold drink, squash	11 615	38	309.73	116.15
**6**	Tea, brewed	9167.5	37	247.77	91.68
**7**	Milk	6110	36	169.72	61.10
**8**	Apple	5000	32	156.25	50.00
**9**	Banana	2752.5	30	91.75	27.53
**10**	Sugar	377.75	22	17.57	3.78
**11**	Margarine	242.5	20	12.13	2.43
**12**	Cold drink, carbonated	7150	19	386.49	71.50
**13**	Egg, fried in sunflower oil	1567.5	18	89.57	15.68
**14**	Beef curry, stew	1787.5	15	123.28	17.88
**15**	Cabbage, cooked	1440	14	102.86	14.40
**16**	Fruit juice	4625	14	330.36	46.25
**17**	Polony	262.5	14	19.44	2.63
**18**	Vegetable curry	572.5	13	45.80	5.73
**19**	Orange	1590	10	159.00	15.90
**20**	Soup powder, prepared with water	247.5	10	24.75	2.48

Table 5 indicates that women consumed a medium FVS with a mean intake of 31.02 different foods across the nine food groups in 7 days; only one person consumed more than 60 foods, indicating a high FVS. The food group with the biggest variety was the cereal, roots and tubers group, with a mean of 6.6 different food items. Fruit and vegetables are divided into three groups: vitamin A rich, other fruit and other vegetable, and each of these had a mean of 3.97, 4.30 and 4.56, respectively. The legumes and nuts group reflected a low score (1.98) over 7 days.

**TABLE 5:** Food variety score mean, standard deviations and the range of scores for the nine food groups (*n* = 100).

Food group	Mean	± Standard deviation	Range of scores
Flesh group diversity	4.51	2.01	0–10
Egg diversity	1.0	0.00	0–1
Dairy products diversity	3.84	2.24	0–10
Cereal, roots and tubers group	6.60	3.00	0–18
Legumes and nuts group	1.98	1.28	0–6
Vitamin A rich fruit and vegetable group	3.97	1.79	0–8
Other fruit diversity	4.30	1.96	0–12
Other vegetable diversity	4.56	2.22	0–12
Fats and oil diversity	2.53	1.14	0–5
Total food items	31.02	11.03	6–62

## Discussion

Pregnancy is a critical period during which maternal nutrient intake and nutritional status impact the health of both the mother and the infant. Various factors play a role in a healthy pregnancy outcome, including good nutrition, appropriate supplementation, smoking habits, drug use, alcohol use and activity levels (Academy of Nutrition and Dietetics 2014). Pregnant women (*N* = 100) who participated in this study were largely in their mid-20s and were relatively well educated, with close to three-quarters holding secondary school qualification. However, nearly two-thirds were unemployed (*n* = 41), with low household income (< R1500/month). Although various other factors can impact birth outcomes, both employment status and household income likely influenced food choices despite the fact that respondents were relatively well educated.

The socioeconomic status of the women indicated that 72.7% of the women had completed secondary school education, which is higher than the finding of the South African National Health and Nutrition Examination Survey 2012 (SANHANES), where 32.8% of the participants had completed high school and 20.2% had completed matriculation (Shisana et al. 2014:64). Although the households seem poor when considering income levels where 67.20% of the households earned less than R1500 per month, a large number of participants reported having a shortage of money to purchase food. The SANHANES found that 45.6% of South Africans were food secure, which was reflected in this study as well (Shisana et al. 2014). The income of women in this study was less than the mean income per household from work in urban informal areas in South Africa, which was R3865 per month as presented in the Living Conditions of Households in South Africa (LCHSA) report 2014 and 2015 (Statistics SA 2015). Furthermore, the SANHANES indicated that 31.8% of women in KZN had a monthly household income of R801 – R3200 and 15.5% of had a household income of less than R800 per month in 2012 (Shisana et al. 2014). In the current study, 23% of the households spent less than R500 per month on food, which was similar to the LCHSA study (2014/2015), which reported that South African household expenditure allocated to food, beverages and tobacco was only 13.75% (Statistics SA 2015). Low income has also been identified as a barrier to a varied diet in a large number of maternal studies in low- and middle-income countries (Kavle & Landry 2018). Even though a large number of households had access to running water (90%) and waste removal services (70%), which serves as a positive socioeconomic indicator, high unemployment and low income may have adversely affected dietary choices of these households.

The results reported here show that 28% of the women in this sample were overweight and 30% were obese. These findings are in line with the 2003 South African National Food Consumption Survey (NFCS) results, where 28% and 27% of adult women in South Africa were found to be overweight and obese, respectively (Labadarios et al. 2008). The study findings are also consistent with the SANHANES report which found that 25.2% and 44% of women in KZN were overweight and obese, respectively (Shisana et al. 2014). The Academy of Nutrition and Dietetics (2014) reiterates the importance for pregnant women to fall within the normal BMI range of 18.5–24.5 and to gain weight within the recommended guidelines set by the Institute of Medicine (IOM) as literature demonstrates that maternal diet and lifestyle choices impact the health of the infant (Rodgers & Yaktine 2013).

The World Health Organization (2012) reported that 35% of women worldwide are overweight, with another one-third being obese, allowing for more women to enter pregnancy with a BMI exceeding 30 kg/m^2^. The infants of obese women typically have a heavier birthweight and are at risk of becoming obese and developing type 2 diabetes as children (World Health Organization 2012). Diet and nutrient intake energy requirements increase during pregnancy to accommodate the increased maternal body mass and foetal growth, which are mainly provided by the macronutrient intakes in the diet. However, the overweight and obesity levels in the early stages of pregnancy are a concern and the large portion sizes recorded for starch-based foods in this study could have impacted increased BMI among women. Carbohydrates are important for the development of the foetal brain and it is recommended that pregnant women consume 175 g carbohydrates a day to meet foetal brain needs for glucose (Brown 2011).

A study by Chen et al. (2017) reported that higher maternal carbohydrate and sugar intake, measured at 26–28 weeks’ gestation, was associated with higher BMI in early childhood. However, it should be noted that a potential contributing factor might have been the gestational age at which BMI was calculated. Ideally, BMI should be calculated prior to pregnancy or as early in the pregnancy as possible. Calculating BMI as late as 24 weeks gestation may have inflated BMI values (Olafsdottir et al. 2006). The mean ± SD carbohydrate intake of the women in this study was 191.82 ± 68.718 g, which is 142% of the recommended DRI. The top 20 food items consumed reflected foods high in carbohydrates and this high intake is also reflected in the FVS, indicating that the women consumed up to 18 different cereals, roots and tubers foods over 7 days. A mean ± SD protein intake of 50.44 ± 20.441 g was recorded in this group of women, with 84% consuming less than the RDA (71 g per day). During pregnancy, protein synthesis shifts more to meeting maternal and foetal protein needs and is used less for energy synthesis (Brown 2011).

Intake of the top 20 foods (Table 3) reflects that protein appeared three times in the diet of the women, with chicken, egg and beef curry intake of 133.00 g, 89.57 g and 123.28 g, respectively, per frequency intake. It is recommended that one portion of protein during pregnancy equals 30 g of meat, 30 g of cheese, 1 egg (± 30 g) or half a cup of cooked dry beans, and six portions can be consumed per day (Nutrition Information Centre University of Stellenbosch 2017b). Therefore, pregnant women in the first trimester of pregnancy should consume 180 g of protein a day. The women in this study consumed a mean of 115 g per day. Maternal micronutrient status is important for a healthy birth outcome of the infant, as the body’s needs, especially for iron, calcium, folic acid and vitamins A, increase (Nutrition Information Centre University of Stellenbosch 2017b). Although fruit and vegetables (apple, banana, cabbage, fruit juice, vegetable curry and orange) featured on the top 20 list, the women still had a per capita intake of 165.73 ± 64.509 g fruit and vegetables, which is 41.43% of the World Health Organization (2003) daily recommended amount of > 400 g, thus indicating a low micronutrient intake.

The need for iron increases substantially during pregnancy, from 8.1 mg to 22 mg EAR per day, and although iron-rich sources were observed twice in the top 20 food intake list as red meat and egg, it only reached a mean ± SD of 10.70 ± 4.175 mg, which was less than 50% of the required amount (National Academy of Sciences, 2016; National Nutrition Information Centre of the University of Stellenbosch 2017b). In South Africa, one out of five women has been found to have poor iron intake, increasing the risk of preterm birth and low birthweight infants (Brown 2011). Calcium is required during pregnancy for foetal skeletal mineralisation and maintenance of maternal bone health and pregnant women require a 1000 mg adequate intake (AI) to retain calcium stores. The women in this study consumed a mean ± SD intake of 308.86 ± 235.238 mg, which is a mere 30.81% of the DRIs, and 98.0% of the women did not meet 100% of the DRIs (National Academy of Sciences 2016). This is a serious problem as calcium is released from the bones when the intake is deficient and when the calcium stores are not replensihed it can contribute to osteoporosis. The top 20 calcium-rich foods consumed by women in the current study reflected milk at number 7 on the list. However, milk was reportedly consumed by only 36/100 participants on a daily basis, with an average portion size of 169.72 mL. Oranges, egg and cabbage were the only other sources providing some calcium intake. No fish or seafood providing calcium supplementation appeared on the food intake list.

The women in this study consumed a mean 270.83 µg of dietary folic acid, which was almost half of the recommended amount. This was in contrast to the SA NFCS data that reported that folate status across the country was adequate in 2005 (Labadarios et al. 2008). Folic acid is an important nutrient, which has been implicated in the prevention of neural tube defects in the foetus. Therefore, women capable of falling pregnant should consume an estimated average requirement (EAR) of 320 µg per day before conceiving and increase this to an EAR of 520 µg dietary folic acid a day once pregnant or alternatively should be on daily folic acid supplementation at 400 mcg (National Academy of Sciences 2016). Good sources of folic acid include wheat, dry beans, cabbage, liver, kidneys, eggs, lentils and yeast (Nutrition Information Centre University of Stellenbosch 2017b) of which only eggs (89.57 g) and cabbage (102.86 g) appear in the top 20 foods per frequency consumed with a very low consumption of legumes and nuts as observed in the FVS.

Vitamin A deficiency (VAD) early in pregnancy can cause malformation of the foetal lungs, urinary tract and heart. Excessive intake of vitamin A during pregnancy is also undesirable as it can cause retinoic acid syndrome in the foetus, a syndrome affecting the ear canals and brain formation and causing heart defects (Brown 2011). The FVS indicates that the women consumed a mean of 3.97 different fruit and vegetables high in vitamin A over the 7 day period. Milk, eggs, fruit juice, vegetable curry and margarine appear in the top 20 food intake list, thus contributing to vitamin A intake. The recommended amount during pregnancy is 550 µg, but in the study group the women consumed a mean ± SD of 643.53 ± 1450.346 µg. As 79% of the women in this group consumed amounts of vitamin A more than the RDA, VAD is a potential concern. The SANHANES indicated a VAD prevalence of 16.4% in women of reproductive age in KZN, representing a moderate public health problem (Shisana et al. 2014).

Apart from carbohydrates and vitamin A, all the nutrients consumed were lower than the recommended daily amounts and the food choices made by the women do not support healthy food choices to ensure that a favourable pregnancy is experienced. More than 50% of the women were overweight or obese in the first trimester of pregnancy, increasing the risk of gestational diabetes and pre-eclampsia, as well as other adverse perinatal outcomes (Athukorala 2010). The current nutrient intake, portion size consumption and nutritional status of the women confer the risk of a poor birth outcome and obesity in early life.

In summary, the majority of women in this study were unemployed, with a reported income of less than R1500 per month. Diets during pregnancy were high in carbohydrates and starches, low in protein and iron and often more than the recommended vitamin A intake. The results of this study highlight the need for patient awareness about nutritional needs during pregnancy. Such education could be accomplished using social media campaigns and through targeted educational programmes. Additional research is needed to promote healthy nutrition during pregnancy, particularly where patient resources are limited.

### Limitations

A limitation of this study was that pregnant women in the South African PHC system have been known to delay prenatal care, with the majority entering care between 8 and 24 weeks gestation. Women who presented early in pregnancy might potentially have been experiencing nausea and vomiting, which may have impacted dietary intake and frequency. Pregnant women in their first trimester (normally through 13 weeks) (WHO 2016) may present with a different BMI compared to women above 14 weeks. An additional limitation of the study was the determination of BMI at as late as 24 weeks’ gestation. The WHO recommendations for weight gain during pregnancy are based on pre-pregnant BMI or early in pregnancy. Many women in this study entered care late and the subsequent BMI calculations may have biased the findings. Even though an experienced midwife confirmed gestational dates, further confirmation by ultrasound was not available, which was a limitation. In addition, this study did not examine perinatal outcomes and maternal characteristics such as parity, gravida and marital status that may be an important area in need of further investigation. Finally, these women represent a lower socioeconomic group and nutrition results reported here cannot be generalised to pregnant women from higher socioeconomic groups.

## Conclusion

Although various factors, such as undesirable weight before pregnancy, maternal illness, medication and lifestyle, can impact perinatal outcomes, nutrition plays a major role in the health of the foetus and the mother. Study findings underscore the need to increase awareness among pregnant women regarding the importance of a balanced diet, including AI of iron and folic acid. Nutrition education for women of childbearing age would be an important component for increasing awareness. Although a nutrition adviser was available at the clinic where the study was conducted, the content of nutrition education should be investigated with regard to information on the availability of fortified foods. Such efforts would promote a diet addressing the nutrient requirements of pregnant women.
